# Theory-Driven Tailoring of the Microenvironment of Quaternary Ammonium Binding Sites on Electrospun Nanofibers for Efficient Bilirubin Removal in Hemoperfusion

**DOI:** 10.3390/polym16111599

**Published:** 2024-06-05

**Authors:** Xingyu Fu, Minsi Shi, Dingyang Chen, Xinyue Zhao, Tingting Jiang, Rui Zhao

**Affiliations:** 1 Key Laboratory of Polyoxometalate and Reticular Material Chemistry of Ministry of Education, Faculty of Chemistry, Northeast Normal University, Changchun 130024, China; fuxingyu@nenu.edu.cn (X.F.); shiminsi@126.com (M.S.); chendy799@nenu.com (D.C.); zxy2022aaa@163.com (X.Z.); 2 School of Chemical Engineering, Northeast Electric Power University, Jilin 132012, China

**Keywords:** electrospinning, grafting, adsorption, bilirubin, hemoperfusion

## Abstract

Efficient adsorbents for excess bilirubin removal are extremely important for the treatment of hyperbilirubinemia. However, traditional adsorbents, such as activated carbons and ion-exchange resins, still suffer from dissatisfactory adsorption performance and poor blood compatibility. Herein, we adopted a rational design strategy guided by density functional theory (DFT) calculations to prepare blood-compatible quaternary ammonium group grafted electrospun polyacrylonitrile nanofiber adsorbents. The calculation analysis and adsorption experiments were used to investigate the structure–function relationship between group types and bilirubin adsorption, both indicating that quaternary ammonium groups with suitable configurations played a crucial role in bilirubin binding. The obtained nanofiber adsorbents showed the bilirubin removal efficiency above 90% even at a coexisting BSA concentration of 50 g L^−1^. The maximum adsorption capacities were 818.9 mg g^−1^ in free bilirubin solution and 163.7 mg g^−1^ in albumin bound bilirubin solution. The nanofiber adsorbents also showed considerable bilirubin removal in dynamic adsorption to reduce the bilirubin concentration to a normal level, which was better than commercial activated carbons. Our study demonstrates the high feasibility of a theory-driven design method for the development of grafted electrospun nanofibers, which have good potential as bilirubin adsorbents in hemoperfusion applications.

## 1. Introduction

Bilirubin is a metabolite of aging red blood cells, and is transported by the blood to the liver for metabolism and excreted through the biliary tract [1]. If the body experiences symptoms such as liver damage or blockage of the biliary tract, excessive production of bilirubin can occur in the blood, resulting in so-called hyperbilirubinemia. Hyperbilirubinemia can lead to jaundice, hepatitis, brain damage, and in severe cases, death. Meanwhile, other complications in patients have been clinically found to be accompanied by a massive accumulation of bilirubin in the human body [2,3]. How to quickly and effectively remove excess bilirubin from blood is an urgent issue that needs to be solved. At present, blood purification therapy has the most therapeutic effect on patients with hyperbilirubinemia in clinical practice [4]. Blood purification therapy mainly includes blood replacement, hemodialysis, and hemoperfusion adsorption [5]. Blood replacement requires a large amount of fresh plasma, which is expensive and difficult in terms of clinical application. Hemodialysis has a good removal effect on small blood poisons; however, it has little effect on the removal of albumin-bound bilirubin. Among them, hemoperfusion adsorption is regarded as a simple, efficient, and practical bilirubin treatment method, which can remove albumin-bound bilirubin to the maximum extent. The core component of the hemoperfusion method is the specific bilirubin adsorbents [6].

**Figure 1:**
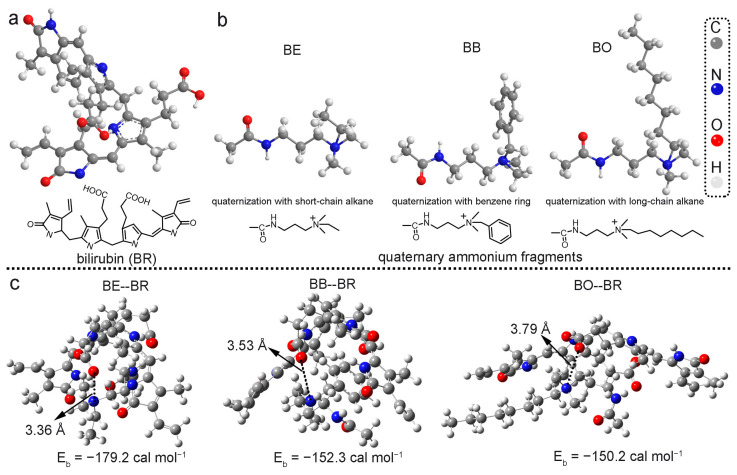
(**a**) Chemical structure of bilirubin. (**b**) Chemical structures of the designed quaternary ammonium groups. (**c**) The optimized adsorption complexes of bilirubin with the three quaternary ammonium groups via density functional theory.

In the past few decades, various adsorbents, including activated carbons [7], polymer resins [8], natural polymers [2], inorganic mesoporous materials [5], etc., have been widely studied in hemoperfusion therapy to remove bilirubin. However, most of them have problems such as low adsorption performance, complex separation, high cost, and poor blood compatibility. Therefore, it is necessary to develop novel adsorption materials with high adsorption efficiency and blood safety for bilirubin adsorption, but this remains challenging. Electrospinning technology is a feasible method to produce continuous one-dimensional nanofibers with the action of a high-voltage electrostatic field [9]. This method exhibits simple equipment, controllable preparation process, abundant raw materials, and a relatively low cost. The obtained nanofibers have high porosity, high pore connectivity, easy separation, and facile modification properties [10]. As a result, electrospun fibers are good candidates for adsorbents in aqueous solution systems [11]. Recently, electrospun fibers have been used as bilirubin adsorbents during hemoperfusion to purify blood. Natural polymers and synthetic polymers have been electrospun for bilirubin adsorption. Ma and co-workers applied electrospun cellulose nanofibers as an affinity membrane, and this nanofiber membrane showed a bilirubin adsorption capacity of 4.0 mg g^−1^ [12]. In Jiao’s group, nanoporous polyethersulfone (PES) fiber mats were prepared via electrospinning and this adsorbent displayed an adsorption capacity of 176.0 mg g^−1^ for bilirubin [13]. They also immobilized bovine serum albumin on electrospun PES fibers, which showed an excellent bilirubin adsorption capacity of 192.4 mg g^−1^ [14]. Moreover, grafted electrospun polyacrylonitrile nanofibers also showed promising bilirubin removal. In our previous work, branched polyethylenimine was grafted on electrospun polyacrylonitrile fibers and they exhibited a maximum adsorption capacity toward albumin-bonded bilirubin of 112.9 mg g^−1^ [15]. Liu and co-workers grafted lysine on polyacrylonitrile electrospun nanofibers. The maximum adsorption capacity toward bilirubin was 64.0 mg g^−1^ [16]. Studies have found that necessary modifications need to be carried out on electrospun fibers before their usage as bilirubin adsorbents. Nevertheless, there has been a lack of systematic research into the interaction strength between functional groups and bilirubin molecules. Investigation of this structure–function relationship is needed. Based on the chemical structure (Figure 1a), bilirubin has anionic groups and conjugated five-membered rings. Moreover, bilirubin is also a hydrophobic molecule [17]. Thus, modifying the electrospun fiber adsorbents with positively charged functional groups with different microenvironments is an effective approach to study the interaction between cationic adsorption sites and bilirubin, which is beneficial to the design desirable adsorbents.

Based on the above reasons, in this work, our proposed method was firstly to utilize quantitative calculations for functional group design, avoiding the consumption of significant human and financial resources [18]. Three quaternary ammonium groups with different microenvironments of ethyl, tolyl, and octyl structures, which considered electrostatic interaction, π−π interaction, and hydrophobic interaction, were designed. The interaction energy of three quaternary ammonium groups on bilirubin was calculated and compared to find the suitable functional groups. To verify the calculations, the designed three quaternary ammonium groups were grafted on the electrospun nanofibers for bilirubin adsorption. Both calculation and experiment results suggested that the quaternary ammonium group with low steric resistance showed high bilirubin affinity. The adsorption rate of the obtained nanofiber adsorbent was faster than that of commercial activated carbon and resin. The obtained maximum adsorption capacities for free bilirubin solution and albumin-bound bilirubin solution were 818.9 mg g^−1^ and 163.7 mg g^−1^, respectively. The nanofiber adsorbents could also remove more than 90% of bilirubin from albumin-bound bilirubin solution. In the simulated hemoperfusion experiments, the designed nanofibers could effectively decrease the bilirubin concentration to a normal level, which was much better than commercial activated carbon. The overall results demonstrate that our designed electrospun nanofiber adsorbent is expected to be used as a high-affinity bilirubin adsorbent in hemoperfusion.

## 2. Materials and Methods

### 2.1. Materials and Instruments

Polyacrylonitrile (PAN, Mw = 150,000 g mol^−1^), bilirubin (98%), and multi-walled carbon nanotubes (CNTs), were purchased from Aladdin Biochemical Technology Co., Ltd., Shanghai, China. *N*,*N*-dimethylformamide (DMF) was purchased from Tiantai Chemical Corporation, Guangzhou, China. *N*,*N*-dimethyl-1,3-propanediamine (98%), bromoethane (99%), benzyl bromide (98%), and 1-bromooctane (98%) were obtained from Energy Chemical, Shanghai, China. All reagents were used without further purification. FT-IR spectra were recorded on a Nicolet iS50 Fourier transform infrared spectrometer from 4000 to 400 cm^−1^. Field emission scanning electron microscopy (SEM, Shimadzu SSX-550, Kyoto, Japan) was used to observe the morphology.

### 2.2. Synthesis of Grafted Electrospun Nanofibers

PAN nanofibers were obtained from the electrospinning process. Briefly, PAN (0.8 g), and CNTs (0.8 g) powders were dissolved in 10 mL of DMF for 8 h with magnetic stirring at 90 °C to form a homogeneous solution. Then, the obtained solution was loaded into a 20 mL plastic syringe with a stainless-steel needle (internal diameter: 0.5 mm) with a distance between the needle tip and the collector of 20 cm and an electrospinning voltage of 16 kV. The flow rate of the solution was controlled at 0.5 mL h^−1^ by a syringe pump. PAN nanofibers were collected from the collector.

The grafting processes were conducted as follows: 500 mg of obtained PAN nanofibers, 14 mL of *N*,*N*-dimethyl-1,3-propanediamine, and 12 mL of water were added into a three-neck flask for reflux process for 3 h. After the reaction mixture was cooled, the aminated PAN nanofibers were washed by water and ethanol in turn, then dried in vacuum oven at 60 °C for 12 h.

The aminated PAN nanofibers were added into ethanol solution containing the quaternary reagent (bromoethane, benzyl bromide, and 1-bromooctane; the weight ratio of quaternary reagent and aminated PAN nanofibers was 10:1). The reaction mixture was stirred for 24 h under reflux. Then, the grafted electrospun nanofibers were filtered and washed repeatedly with ethanol and water. After washing, the products were dried in a vacuum oven at 60 °C for 12 h.

### 2.3. Bilirubin Adsorption Experiments

Due to the high sensitivity of bilirubin to light, all solution preparation and adsorption experiments were carried out in the dark. To obtain a homogeneous bilirubin solution, a certain amount of bilirubin was initially dissolved in a small volume of dimethyl sulfoxide and 0.1 M Na_2_CO_3_ solution, and then diluted with phosphate buffer saline (PBS, pH = 7.4) to obtain the desired concentration. The concentration of bilirubin was determined using a UV–visible spectrophotometer. Each adsorption experiment was conducted in triplicate to obtain reproductive results. The amount of adsorbed bilirubin was analyzed according to the following equation:

(1) $$q\;\left(\mathrm{mg}/g\right)=\frac{\left(C_{0}-C_{e}\right)V}{W}$$

where C_0_ and C_e_ are the initial concentration and equilibrium concentration of bilirubin in the test solution (mg L^−1^), V is the volume of the test solution (L), and W is the mass of the adsorbent (g).

For the dynamic adsorption experiment, an appropriate amount of nanofiber adsorbent was placed into the adsorption column, and the dynamic adsorption was performed by the power pump at an appropriate speed for 4 h. At a certain time, the bilirubin concentration was determined.

### 2.4. Blood Compatibility Experiments

Hemolysis Assay: Hemolytic ability was determined by incubating the nanofibers with red blood cells at 37 °C for 1 h. Then, 1 mL rat blood was placed in the centrifuge tube and centrifuged at 3000 rpm for 10 min. The serum (upper layer) was discarded, and the blood cells (under layer) were washed three times with PBS solution and then diluted to 10 mL. Fiber materials were washed with PBS and then fibers with different amounts (10, 20, 60, and 100 mg, respectively) were added into the as-mentioned suspension. Deionized water and PBS were used as positive and negative controls, respectively. After incubation at 37 °C for 1 h, the mixture was centrifuged at 3000 rpm 10 min and the absorbance of the supernatant solution at 541 nm was recorded by UV–vis spectroscopy. The hemolysis rate was calculated using the following equation:

(2) $$\mathrm{Hemolysis}\;\mathrm{rate}\;\left(\%\right)=\frac{{\mathrm{OD}}_{s}-{\mathrm{OD}}_{\mathrm{nc}}}{{\mathrm{OD}}_{\mathrm{pc}}-{\mathrm{OD}}_{\mathrm{nc}}}\times 100\;$$

in which OD_s_, OD_pc_, and OD_nc_ are the absorbances of the sample, positive control, and negative control, respectively.

Coagulation time tests: The activated partial time (APTT), thrombin time (TT), and prothrombin time (PT) were used to evaluate the anticoagulation property. Firstly, different amounts of fiber materials were put into 24-well plate, and the physiological saline was added into the plates for immersion overnight and then incubated at 37 °C for 1 h. After removing the physiological saline, 100 µL fresh platelet-poor plasma (PPP) was introduced and incubated with the nanofibers at 37 °C for 20 min. Platelet-poor plasma (PPP) could be obtained by centrifuging the whole anticoagulant blood at 4000 rpm for 15 min. For APTT measurement, 100 μL of APTT agent was added to the wells at 37 °C and incubated at 37 °C for another 5 min. Then, 100 μL of 0.025 M CaCl_2_ was added. Then, the coagulation time was measured as an APTT value. For the TT test, 100 μL of thrombin agent (incubated 10 min at 37 °C before use) was added to the wells, and then TT values were measured. To test PT, 100 µL Thromborel S (incubated 10 min before use) was added to the wells at 37 °C and further incubated at 37 °C for 2 min, and then the PT values were measured. Pure PPP without samples was the control group. All measurements were carried out six times.

Platelet adhesion test: To study platelet adhesion, the fiber membranes (1 cm × 1 cm) were placed in a 24-well cell culture plate. One milliliter of fresh platelet-rich plasma (PRP) was dropped in each well and then incubated at 37 °C for 2 h. The PRP was removed with an aspirator, and the membrane was rinsed three times with normal saline, and then the adhered platelets were fixed with 2.5 wt% glutaraldehyde in normal saline at 4 °C for 12 h. Finally, the samples were washed with normal saline, and then dehydrated with a series of ethanol/normal saline mixtures of increasing ethanol concentration (0, 25, 50, 75, and 100 wt% ethanol, 15 min in each mixture) and then dried at room temperature. The platelet adhesion was observed using scanning electron microscopy.

### 2.5. Computational Method

The interaction energies between the quaternary ammonium fragments and bilirubin were evaluated using quantum chemical calculations. The 3D structures of the fragments were built with GaussView5.0.8, and bilirubin 3D structure was downloaded from the pubchem website. All calculations were carried out using the density functional theory (DFT) with the B3LYP function [19,20], as implemented in the Gaussian 09 program [21]. All geometry structures were optimized with the 6-31G(d,p) basis set. On the basis of optimized geometries, the best binding structures of bilirubin with the quaternary ammonium fragments were searched with the AutoDock Vina program [22], in which all parameters are default. Following this, all binding structures were further optimized using B3LYP with 6-31G(d,p) basis set. To obtain more accurate energies, single-point calculations were performed based on these optimized geometries with the 6-311++G(2d,2p) basis set on all atoms. All binding energies (BEs) were calculated to evaluate the adsorption strengths of bilirubin in the different quaternary ammonium fragments. Basis set superposition error (BSSE) was considered in the calculations of binding energies.

## 3. Results

### 3.1. Design and Theoretical Calculations

Cationic quaternary ammonium groups are the common functional groups used for bilirubin adsorption in the previous literature [23] and commercially available adsorbents. Moreover, the chemical environments surrounding the quaternary ammonium sites are also important for bilirubin (BR) binding [17]. In this work, three quaternary ammonium groups with different quaternary structures were selected as the theoretical models to assess their binding ability toward bilirubin molecules through performing density functional theory (DFT) calculations. The different quaternary structures were ethyl, tolyl, and octyl building units, respectively (Figure 1b). The ethyl quaternary structure (BE) could only provide electrostatic interaction. The octyl quaternary structure (BO) could provide electrostatic interaction and hydrophobic interaction. The tolyl quaternary structure (BB) could provide electrostatic interaction, π−π interaction, and hydrophobic interaction. As a result, the influence of chemical structures of quaternary ammonium groups involving the multiple interactions in the bilirubin adsorption was comprehensively investigated. The optimized adsorption geometries of these functional groups toward bilirubin are displayed in Figure 1c. Interestingly, we found that the calculated binding energy (E_b_) of BE--BR (−179.2 kcal mol^−1^), which only involved electrostatic interaction, was higher than that of the other two systems (BB--BR (−152.3 kcal mol^−1^) and BO--BR (−150.2 kcal mol^−1^)). The results indicated that electrostatic interaction was the main driving force for bilirubin binding. Though other van der Waals forces (π−π interaction and hydrophobic interaction) were also attributed to bilirubin adsorption, the geometry of the quaternary ammonium group was more important. It was observed that the bond distance belonging to the electrostatic interaction of BE--BR was lower than that of BB--BR and BO--BR. Introducing a large quaternary structure, such as tolyl and octyl units, significantly increased the steric hindrance. The calculated data suggested that quaternary ammonium group with low steric hindrance showed the strongest binding affinity toward bilirubin, which guided the design of more efficient adsorbents for bilirubin.

### 3.2. Preparation and Characterization of Three Electrospun Nanofiber Adsorbents

**Figure 2:**
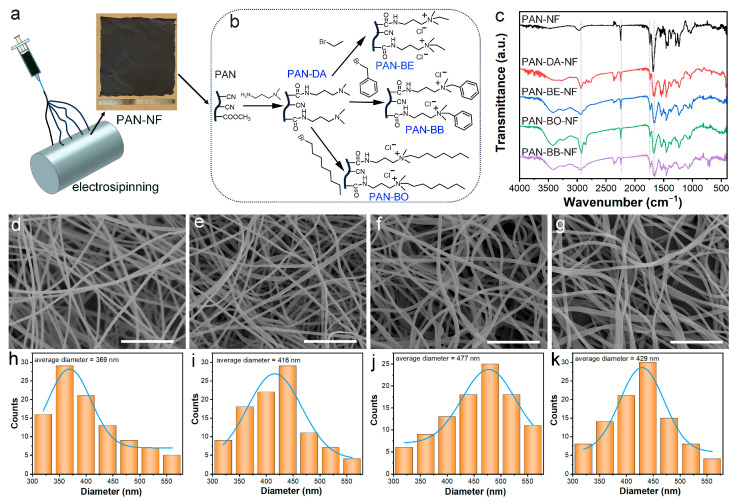
(**a**) Schematic illustration of the electrospinning process. (**b**) The grafting routes of the quaternary ammonium group modified electrospun nanofibers. (**c**) FT-IR spectra of the obtained fibers. SEM images and diameter distributions of PAN-NF (**d**,**h**), PAN-BE-NF (**e**,**i**), PAN-BO-NF (**f**,**j**), and PAN-BB-NF (**g**,**k**) (the scale bar is 10 µm).

Based on the theoretical analysis and preparation feasibility, the above three quaternary ammonium groups were grafted onto electrospun nanofibers. Electrospun polyacrylonitrile (PAN) nanofibers (PAN-NFs) were selected as the substrates because cyano groups of PAN-NFs have high chemical reactivity for grafting. To improve the mechanical strength of the PAN-NFs, carbon nanotubes served as a reinforcement component in the electrospinning solution (Figure 2a). A simple three-step approach was applied to prepare the three quaternary ammonium grafted electrospun nanofibers (Figure 2b). First, PAN-NF was reacted with *N*,*N*-dimethyl-1,3-propanediamine to introduce tertiary amine on the nanofibers, yielding PAN-DA-NF. Then, PAN-DA-NF was reacted with different quaternary reagents (bromoethane, benzyl bromide, and 1-bromooctane). To reduce the toxicity, counter-balancing Br^−^ ions were ion-exchanged by Cl^−^ ions, finally yielding the targeted PAN-BE-NF, PAN-BB-NF, and PAN-BO-NF, respectively. These grafted quaternary ammonium groups were consistent with the fragments in theoretical calculations.

The chemical structures of the prepared nanofibers were characterized by FT-IR spectra. As shown in Figure 2c, PAN-NF displayed typical characteristic peaks of polyacrylonitrile. Peaks at 2247 cm^−1^ and 1738 cm^−1^ were assigned to the vibrations of cyano groups and carbonyl groups of methacrylate copolymers [9]. The peaks at 2930 cm^−1^ and 2859 cm^−1^ belonged to the C-H stretching vibration of alkane groups [24]. After the amination reaction, the intensities of peaks at 2247 cm^−1^ and 1738 cm^−1^ were reduced in the spectrum of PAN-DA-NF. Moreover, PAN-DA-NF showed a new strong absorption peak at 1659 cm^−1^, which was due to the stretching of the C=O amide group [3]. These results demonstrated the hydrolysis of cyano and methacrylate groups, and the amidation reaction occurred between the amino group of *N*,*N*-dimethyl-1,3-propanediamine and the hydrolyzed carboxyl group. After the quaternization reaction, the spectrum of PAN-BE-NF showed few changes compared with that of PAN-DA-NF. However, the peak intensities at 2930 cm^−1^ and 2859 cm^−1^ were evidently enhanced for PAN-BO-NF, owing to the grafted long alkyl chain. The spectrum of PAN-BB NF exhibited a bending vibration of the benzene ring at 698 cm^−1^ and 733 cm^−1^ [25]. The degree of modification of grafted nanofibers can be expressed by weight gain (%) and functionality (mmol g^–1^). Their expressions are showed as follows:

(3) $$\mathrm{Weight}\;\mathrm{gain}\;\left(\%\right)=\frac{W_{m}-W_{0}}{W_{0}}\times 100$$

(4) $$\mathrm{Functionality}=\frac{W_{m}-W_{0}}{W_{m}M}$$

where W_0_ and W_m_ represent the weight of the nanofibers before and after grafting, respectively, and M is the molecular weight caused by functional organic molecules. Correspondingly, the weight gain rate and functional group grafting rate were calculated, and each grafting rate of the quaternary ammonium groups was stable at about 1.8 mmol g^−1^ (Table 1), suggesting that the content of quaternary ammonium groups in the three nanofibers was nearly the same. Combined with FT-IR results, it was proved that different quaternary ammonium groups were grafted onto the electrospun PAN nanofibers successfully. The morphology of the nanofibers was observed using a scanning electron microscope (SEM). All the grafted nanofibers maintained a good fibrous shape, and a large number of pores existed among the fibers (Figure 2d–g), which made them suitable for adsorption applications. As shown in Figure 2h–k, the average diameter of PAN-NF was 369 nm. After the grafting reaction, due to the grafted groups and swelling, the fibers became more disordered and the average diameter increased.

### 3.3. Bilirubin Adsorption Experiments

**Figure 3:**
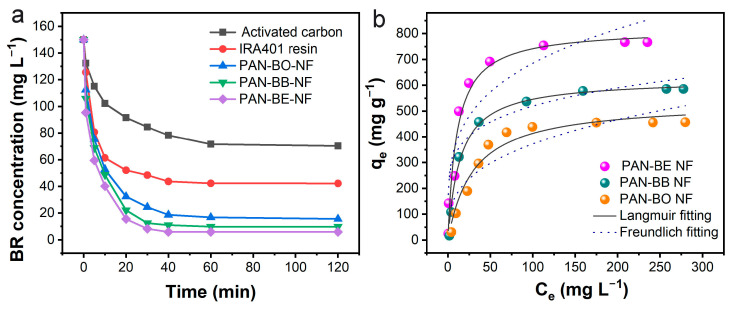
(**a**) Bilirubin (BR) removal versus contact time by different adsorbents (C_BR_ = 150 mg L^−1^, adsorbent dosage = 1 g L^−1^, pH = 7.4, temperature = 37 °C, shaking speed = 120 rpm) and (**b**) bilirubin removal versus contact time by different nanofiber adsorbents (C_BR_ = 20~350 mg L^−1^, adsorbent dosage = 0.1 g L^−1^, pH = 7.4, temperature = 37 °C, shaking speed = 120 rpm).

We conducted a comprehensive examination of the adsorption performance of the obtained grafted electrospun nanofibers toward bilirubin. In order to understand the adsorption rate and saturation adsorption time, the adsorption kinetics were studied. As can be seen from the kinetic curve shown in Figure 3a, the results showed that the PAN-BE-NF displayed the fastest adsorption rate and most efficient adsorption ability for free bilirubin. In the initial concentration of 150 mg L^−1^, the saturation equilibrium time was reached at 40 min, and the residual bilirubin concentration was lower than the normal bilirubin level of the human body (10 mg L^−1^). The equilibrium time for PAN-BB-NF and PAN-BO-NF was 60 min and 120 min, respectively, and their residual concentrations all exceeded 10 mg L^−1^. However, based on the curves, the adsorption performance of the three electrospun nanofibers were all better than the commercial activated carbon and ion-exchange resin. This was because the abundant pores among the fibers were beneficial to the bilirubin molecule diffusion, which accelerated the adsorption rate.

In order to further directly compare the adsorption capacity of the nanofibers, isothermal experiments of free bilirubin adsorption were carried out at the different initial bilirubin concentrations (Figure 3b). Two classical isotherm models, Langmuir and Freundlich equations, were used to describe the equilibrium properties of adsorption. The fitting curves and R^2^ results indicated that these three equilibrium processes were more suitable to be described by the Langmuir model, suggesting that the adsorption sites on the fiber surfaces were uniformly distributed, and the adsorption process could be considered as monolayer adsorption [26]. As summarized in Table 2, PAN-BE-NF had the largest adsorption capacity for free bilirubin uptake, with a maximum adsorption capacity up to 818.9 mg g^−1^. This value was much higher than PAN-BB-NF (623.8 mg g^−1^) and PAN-BO-NF (536.8 mg g^−1^). PAN-BE-NF’s quaternary ammonium groups showed low steric hindrance and high binding energy, resulting in high bilirubin uptake. The adsorption kinetics and isotherms results agreed with the theory calculation analysis, suggesting that the theory-driven method was an effective way to rationally design the adsorbents and avoid the trial-and-error process.

**Figure 4:**
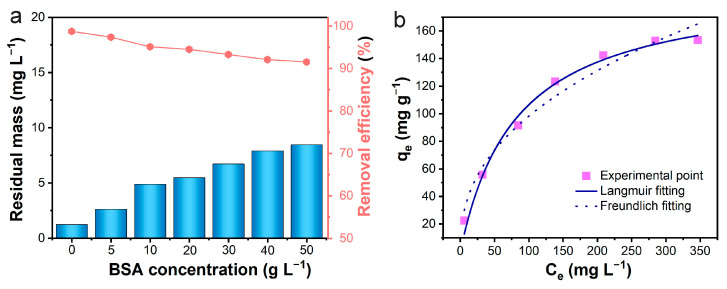
(**a**) Experimental results of adsorption of bilirubin under the coexistence of BSA (C_BR_ = 100 mg L^−1^, adsorbent dosage = 3 g L^−1^, pH = 7.4, temperature = 37 °C, shaking speed = 120 rpm). (**b**) Adsorption isotherm of PAN-BE-NF toward albumin-bound bilirubin (C_BR_ = 25~500 mg L^−1^, C_BSA_ = 50 g L^−1^, adsorbent dosage = 1 g L^−1^, pH = 7.4, temperature = 37 °C, shaking speed = 120 rpm).

Because PAN-BE-NF had the best bilirubin adsorption performance among the three nanofibers, the following experiments about the practical applications focused on PAN-BE-NF. In the blood, the albumin–bilirubin complex is the existing form that interferes with the bilirubin removal by the adsorbents [1]. To simulate the blood environment, bovine serum albumin (BSA) was added into the above bilirubin solution to investigate the effect of albumin on bilirubin adsorption. In the presence of BSA at different concentrations (0–50 g L^−1^), the adsorption efficiency of PAN-BE-NF for bilirubin was above 90% even at a BSA concentration of 50 g L^−1^, and the residual concentration of bilirubin was lower than 10 mg L^−1^ (Figure 4a). The isothermal experiment of the adsorption of albumin-bound bilirubin was also studied (Figure 4b). The concentration of BSA in all albumin-bound bilirubin solutions was fixed at 50 g L^−1^. The fitting curves and R^2^ results (Figure 4b and Table 3) show that the equilibrium process is more suitable to be described by the Langmuir model, and the maximum adsorption capacity of PAN-BE-NF fibers for albumin-bound bilirubin could reach up to 163.7 mg g^−1^. Although the adsorption capacity showed a dramatic decrease owing to competition from BSA, this adsorption capacity for albumin-bound bilirubin still surpassed many reported bilirubin adsorbents (Table S1), suggesting that PAN-BE-NF exhibited a good ability to resist albumin interference.

### 3.4. Blood Compatibility Test

**Figure 5:**
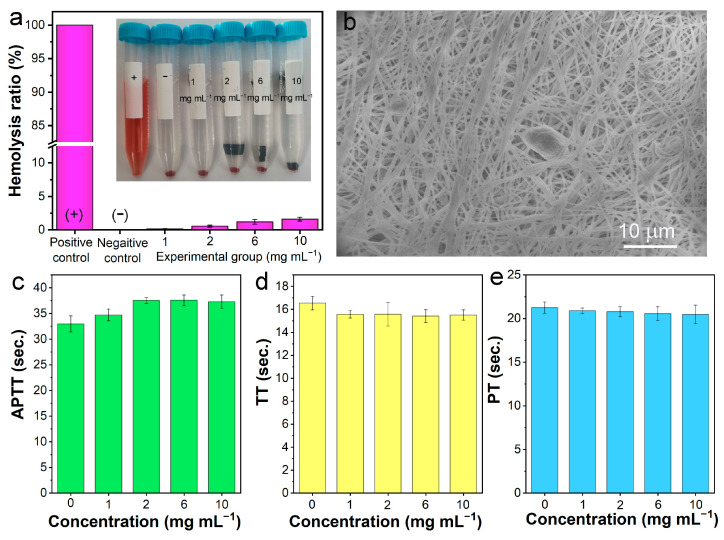
(**a**) Hemolysis test for PAN-BE-NF, in which deionized water and phosphate buffer saline were used as positive and negative controls, respectively. (**b**) SEM images of platelet adhesion on PAN-BE-NF. (**c**) APTT experiment, (**d**) TT experiment, and (**e**) PT experiment results.

For blood purification materials, due to the direct contact with blood, blood compatibility is very important. In this study, the blood compatibility of PAN-BE-NF was characterized by a hemolysis test, platelet adhesion, activated partial thromboplastin time (APTT), plasma thrombin time (TT), and plasma prothrombin time (PT). The hemolysis test is a visual indicator used to evaluate blood compatibility, and materials with a hemolytic value of <5% are considered to be safe as blood-contact materials [15]. The results in Figure 5a indicate that PAN-BE-NF had almost no hemolytic activity, and the hemolysis rate of PAN-BE-NF, even with a concentration of 10 mg mL^−1^, did not exceed 5%. SEM imaging was used to observe the nanofibers after the platelet adhesion experiment, and it was found that fewer platelets adhered to the nanofiber membrane (Figure 5b), indicating its low blood coagulation effect. At the same time, the coagulability of the fiber material upon contact with blood was measured (Figure 5c–e). APTT is the activated partial thromboplastin time used to assess the activity of inherent and common clotting pathways. TT is the plasma thrombin time, which refers to the time required for plasma fibrinogen to be converted into fibrin after adding “standardized” thrombin to the tested plasma. PT is the plasma prothrombin time, which can reflect the function of the exogenous coagulation system, and its adsorption level is used to evaluate the exogenous and common coagulation pathways. Compared with the control group, the APTT activity of PAN-BE-NF showed only a slight increase, and the TT and PT activities did not significantly decrease. These results confirmed that PAN-BE-NF was safe in the application of blood purification.

### 3.5. Dynamic Bilirubin Adsorption

**Figure 6:**
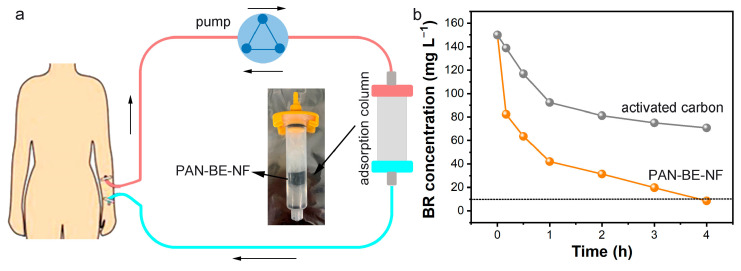
(**a**) Schematic diagram of dynamic cyclic adsorption experiment. (**b**) Experimental results of dynamic adsorption of PAN-BE NF and activated carbon toward albumin-bound bilirubin (C_BR_ = 150 mg L^−1^, mass of adsorption column = 1.0 g, volume of bilirubin solution = 500 mL, flow rate = 100 mL min^−1^).

To simulate the real hemoperfusion process, a dynamic bilirubin adsorption experiment was conducted. PAN-BE-NF was placed into the adsorption column (Figure 6a), and bilirubin solution with 50 g L^−1^ of BSA was forced through the column. The adsorption effect of PAN-BE-NF was much more efficient than that of commercial activated carbon in the circulation system (Figure 6b). PAN-BE-NF could reduce the bilirubin concentration to a normal level of 10 mg L^−1^ within 4 h. However, at this time point, the residual concentration by activated carbon was 70.8 mg L^−1^. The obtained results indicated that the adsorption effect of PAN-BE-NF was still considerable in the state of dynamic adsorption, and PAN-BE-NF possessed potential in practical hemoperfusion application.

## 4. Conclusions

In conclusion, we reasonably designed and synthesized quaternary ammonium group grafted electrospun nanofibers for the efficient adsorption of bilirubin. With the aid of theoretical calculations, we forecasted the effective functional group as the grafting component. Both theoretical analysis and experiment data indicated that the quaternary ammonium group with suitable structural configuration was attributed high bilirubin binding. As a result, the designed nanofibers showed faster bilirubin uptake than commercial activated carbon and ion-exchange resin. The obtained maximum adsorption capacity from free bilirubin was up to 818.9 mg g^−1^. In further experiments, in the presence of different concentrations of BSA, the removal rate of bilirubin was still above 90%, and the concentration of residual bilirubin was lower than 10 mg L^−1^. The maximum adsorption capacity toward albumin-bound bilirubin was 163.7 mg g^−1^, which was higher than most of the reported bilirubin adsorbents. In the dynamic adsorption test, obtained nanofibers also showed better performance than activated carbon, which could reduce the bilirubin concentration to below 10 mg L^−1^. In addition, the blood compatibility indicated the safety of as-prepared nanofibers as hemoperfusion adsorbents. Based on the obtained results, the calculation-guided strategy adopted could offer a universally applicable paradigm for the construction of bilirubin adsorbent in hemoperfusion.

## Figures and Tables

**Table 1:** Modification degree of different nanofibers.

Sample	Weight Gain (%)	Functionality (mmol g^–1^)
PAN-NF	--	--
PAN-DA-NF	25.30%	1.96
PAN-BE-NF	5.50%	1.79
PAN-BB-NF	19.80%	1.82
PAN-BO-NF	25.70%	1.81

**Table 2:** Langmuir and Freundlich constants of different nanofibers for free bilirubin.

Isotherm Model	PAN-BE-NF	PAN-BB-NF	PAN-BO-NF
Langmuir isotherm			
q_max_ (mg g^−1^)	818.9	623.8	536.8
b (L mg^−1^)	0.096	0.07	0.033
R^2^	0.9662	0.9886	0.9561
Freundlich isotherm			
K_F_	210.9	222.9	83.2
n	3.91	5.44	3.07
R^2^	0.8452	0.8782	0.7982

**Table 3:** Langmuir and Freundlich constants of different nanofibers from albumin-bound bilirubin.

Isotherm Model	PAN-BE-NF
Langmuir isotherm	
q_max_ (mg g^−1^)	163.7
b (L mg^−1^)	0.012
R^2^	0.9868
Freundlich isotherm	
K_F_	14.4
n	2.4
R^2^	0.9281

## Data Availability

Data are contained within the article.

## Supplementary Materials

The following supporting information can be downloaded at: https://www.mdpi.com/article/10.3390/polym16111599/s1, Additional experimental section; Table S1: Comparison of bilirubin adsorption results in the presence of BSA. References [15,27,28,29,30,31] are cited in the supplementary materials.
