# Supplementary material for: Theory-Driven Tailoring of the Microenvironment of Quaternary Ammonium Binding Sites on Electrospun Nanofibers for Efficient Bilirubin Removal in Hemoperfusion
Source: Polymers (Basel). 2024 Jun 5;16(11):1599. doi: 10.3390/polym16111599 (PMC11174833; doi:10.3390/polym16111599)
Supplement: Supplementary file 1 [file polymers-16-01599-s001.zip › polymers-3028138-supplementary.pdf]

Supporting Information

Theory-Driven Tailoring of the Microenvironment of Quaternary Ammonium Binding Sites on Electrospun Nanofibers for Efficient Bilirubin Removal in Hemoperfusion

Xingyu Fu [1,†], Minsi Shi [1,†], Dingyang Chen [1], Xinyue Zhao [1], Tingting Jiang [2,*] and Rui Zhao [1,*]

1 Key Laboratory of Polyoxometalate and Reticular Material Chemistry of Ministry of Education, Faculty of Chemistry, Northeast Normal University, Changchun 130024, China; fuxingyu@nenu.edu.cn (X.F.); shiminsi@126.com (M.S.); chendy799@nenu.com (D.C.); zxy2022aaa@163.com (X.Z.)

2 School of Chemical Engineering, Northeast Electric Power University, Jilin 132012, China

* Correspondence: jiangting_chem@neepu.edu.cn (T.J.); zhaor814@nenu.edu.cn (R.Z.)

† These authors contributed equally to this work.

Experimental section

Bilirubin adsorption kinetics. 50 mg of the adsorbents were added into 50 mL bilirubin solution (150 mg L^{-1}). The mixture was shaken for 2 h at 37 °C. At appropriate time intervals, aliquots (1 mL) were taken from the mixture for bilirubin concentration analysis.

Bilirubin adsorption isotherms. 5 mg of the adsorbents were added into 50 mL bilirubin solutions with concentrations ranging from 20 to 350 mg L^{-1}. After adsorption equilibrium, the remaining bilirubin concentrations were analyzed and the adsorption capacities were calculated. The isotherm data were analyzed by two isotherm models, namely Langmuir and Freundlich, whose linear equations are expressed as follows:

Langmuir isotherm (homogeneous and monolayer adsorption):

$$\frac{C_e}{q_e} = \frac{C_e}{q_m} + \frac{1}{bq_m} \qquad (1)$$

Freundlich isotherm (heterogeneous and multilayer adsorption):

$$\log q_e = \log K_F + \frac{1}{n}\log C_e \qquad (2)$$

where q_e is the equilibrium adsorption capacity (mg g^{-1}), C_e is the equilibrium concentration (mg L^{-1}), and q_m and b are Langmuir constants related to maximum adsorption capacity and binding energy, respectively; K_F and n are empirical constants that indicate the Freundlich constant and heterogeneity factor, respectively.

The effect of albumin on bilirubin adsorption. 60 mg of nanofiber adsorbent was added into 20 mL bilirubin solution (100 mg L^{-1}) with different bovine serum albumin (BSA) concentration (0-50 g L^{-1}). Different content of BSA was directly added to the bilirubin solution and stirred to dissolve completely before use. After adsorption equilibrium, the remaining bilirubin concentrations were analyzed and the removal efficiencies were calculated. For adsorption isotherms from albumin-bound bilirubin, 20 mg of the adsorbents were added into 20 mL bilirubin/BSA blending solutions (BSA content: 50 g L^{-1}) with bilirubin concentrations ranging from 25 to 500 mg L^{-1}. After adsorption equilibrium, the remaining bilirubin concentrations were analyzed and the adsorption capacities were calculated.

Blood compatibility experiments

Preparation of blood samples. Fresh blood, obtained from a healthy wister Rat, was mixed with the anticoagulant (3.8% citrate, with a 1:9 ratio of anticoagulant to blood) to get the fresh whole anticoagulant blood. All the animal experiments were conducted in compliance with the guidelines for the care and use of laboratory animals from the National Institutes of Health, and authorize by the Ethics Committee of the Changchun University of Chinese Medicine (ethical permission letter number: 20180094). The authors thank Dr. Yumei Li of the Changchun University of Chinese Medicine (Jilin, China) for blood compatibility tests. The whole anticoagulant blood was centrifuged at 1500 rpm for 15 min, and the resultant supernatant solution is the platelet rich plasma (PRP). Platelet-poor plasma (PPP) was obtained by centrifuging the whole anticoagulant blood at 4000 rpm for 15 min.

Table S1 Comparison of bilirubin adsorption results in the presence of BSA.

Adsorbents	BSA concentration ($g\ L^{-1}$)	Adsorption capacity ($mg\ g^{-1}$)	Ref.
Col-PEI microsphere	50	31.5	[1]
PVA-co-PE	40	37.6	[2]
3D nanofiber sponge	Plasma	25.3	[3]
bPEIPANFM	50	112.9	[4]
m-NpC	40	72.4	[5]
3D-pGR	40	126.1	[6]
PAN-BE-NF	50	163.7	This work

References

1. Zhou, W.; Hu, W.; Zhan, Q.; Zhang, M.; Liu, X.; Hussain, W.; Yu, H.; Wang, S.; Zhou, L. Novel hemoperfusion adsorbents based on collagen for efficient bilirubin removal–A thought from yellow skin of patients with hyperbilirubinemia. *Int. J. Biol. Macromol.* **2023**, *253*, 127321.

2. Wang, W.; Zhang, H.; Zhang, Z.; Luo, M.; Wang, Y.; Liu, Q.; Chen, Y.; Li, M.; Wang, D. Amine-functionalized PVA-co-PE nanofibrous membrane as affinity membrane with high adsorption capacity for bilirubin. *Colloids. Surf. B*. **2017**, *150*, 271-278.

3. Yuan, Z.; Li, Y.; Zhao, D.; Zhang, K.; Wang, F.; Wang, C.; Wen, Y. High efficiency 3D nanofiber sponge for bilirubin removal used in hemoperfusion. *Colloids. Surf. B*. **2018**, *172*, 161-169.

4. Zhao, R.; Li, Y.; Li, X.; Li, Y.; Sun, B.; Chao, S.; Wang, C. Facile hydrothermal synthesis of branched polyethylenimine grafted electrospun polyacrylonitrile fiber membrane as a highly efficient and reusable bilirubin adsorbent in hemoperfusion. *J. Colloid. Interf. Sci*. **2018**, *514*, 675-685.

5. Ma, C.; Gao, Q.; Zhou, J.; Chen, Q.; Han, B.; Xia K.; Zhou, C. Facile one-pot synthesis of magnetic nitrogen-doped porous carbon for high-performance bilirubin removal from BSA-rich solution. *RSC Adv*. **2017**, *7*, 2081.

6. Ma, C.F.; Gao, Q.; Xia, K.S.; Huang, Z.Y.; Han, B.; Zhou, C.G. Three-dimensionally porous graphene: A high-performance adsorbent for removal of albumin-bonded bilirubin. *Colloids. Surf. B*. **2017**, *149*, 146-153.
